# Oxidation and Wear Behaviors of GH3039 Nickel-Based Alloy After Borochromizing

**DOI:** 10.3390/ma19071454

**Published:** 2026-04-05

**Authors:** Lairong Xiao, Haitao Dong, Jiarui Li, Shaofu Xu, Yuxiang Jiang, Zhenwu Peng, Xiaojun Zhao, Zhenyang Cai

**Affiliations:** 1 School of Materials Science and Engineering, Central South University, Changsha 410083, China; 2 State Key Laboratory of Powder Metallurgy, Central South University, Changsha 410083, China

**Keywords:** GH3039, Cr-rich boride layer, tribological performance, wear mechanism, high-temperature oxidation resistance

## Abstract

GH3039 nickel-based alloy, as a key material for thermocouple protection tubes, is susceptible to wear and oxidation failure in high-temperature kiln environment. To address this, boronized, chromized and borochromized coatings were prepared on GH3039 substrate, and the friction-wear properties and high-temperature oxidation resistance of both the substrate and the coatings were systematically characterized. The results show that the borochromized coating, benefiting from the synergistic effect of its relatively high surface hardness and the boric acid lubricating film formed during the wear process, reduces the wear rate by 84.07% (to 1.44 × 10^−5^ mm^3^·N^−1^·m^−1^). Meanwhile, it exhibits the optimal oxidation resistance due to its dense Cr-rich layer, which can inhibit oxygen diffusion and supply chromium for protective Cr_2_O_3_ film. After 100 h of oxidation at 950 °C, its oxidation weight gain is reduced by 78.68% compared with the boronized sample (to 1.20 mg/cm^2^).

## 1. Introduction

Thermocouple protection tubes are critical structural components of kilns, primarily failing through two dominant mechanisms during service: high-temperature atmospheric oxidation and wear induced by slag impact or contact fatigue. Such failures lead to thermocouple measurement malfunction or even complete component scrapping, necessitating frequent furnace shutdowns for replacement, and thus severely restricting industrial production efficiency [1,2,3]. GH3039, a single-phase austenitic solid-solution strengthened nickel-based alloy, is the mainstream material for thermocouple protection tubes due to its excellent high-temperature strength, thermal fatigue resistance, and corrosion resistance at 850 °C [4,5,6]. However, its inherent surface hardness (below 300 HV) results in poor wear resistance [7]. This is particularly problematic when exposed to furnace slag or other hard particles, which can cause cracking and failure. To address this limitation, boronizing is a well-established surface modification technique to improve the tribological properties of nickel-based alloys [8,9,10,11]. For instance, Wu et al. [12] successfully prepare a 24 μm-thick boronized coating on Inconel 718 alloy, achieving a maximum hardness of 1345.9 HV. Boronizing significantly reduces the friction coefficient and wear rate of the alloy by two orders of magnitude. Notably, silicon-containing boronizing media tend to form a porous nickel silicide layer during the boronizing process. This layer not only degrades the tribological properties of the boronized coating but also hinders the growth of the boride layer. Therefore, silicon-free boronizing media are recommended as an alternative [13]. Günen et al. [14] investigated the effect of boronizing on the high-temperature oxidation resistance of Nimonic 80A alloy, revealing that the liquid B_2_O_3_ formed by the oxidation of the boride layer in the short term can reduce oxygen penetration and exert a self-healing effect on materials with microcracks. Nevertheless, prolonged exposure to temperatures exceeding 800 °C causes the volatilization of B_2_O_3_, creating internal oxidation channels. Consequently, developing multifunctional composite coatings with excellent wear resistance of boronized coating and superior high-temperature oxidation resistance has become an urgent engineering challenge.

To enhance both wear resistance and high-temperature oxidation resistance, common strategies include the addition of elements such as Cr, Al and Si. These elements can form continuous and dense oxide films at elevated temperatures, serving as core alloying elements to enhance the high-temperature oxidation resistance of coatings. Lei et al. [15] and Tang et al. [16] conducted boroaluminizing treatment on Inconel 718, demonstrating that the resulting coating improved both the tribological properties and high-temperature oxidation resistance of the alloy. Compared to Al and Si, Cr demonstrates better compatibility with the Ni matrix. Chromizing treatment increases the alloy’s surface chromium concentration, promoting the formation of a denser chromium oxide film during subsequent oxidation and thereby enhancing the high-temperature oxidation resistance of the single boronized coating. Furthermore, B and Cr can form stable chromium borides which further enhance the coating’s hardness and wear resistance.

The preparation of boron-chromium composite coatings primarily includes borochromizing and two-step pack cementation processes. However, during borochromizing, the strong interaction between B and Cr atoms tends to form chromium borides within the pack powder, which severely hinders uniform coating growth. Kheyrodin et al. [17,18,19] confirmed that the treatment sequence in the two-step pack cementation process critically influences coating performance. Specifically, a pre-boronizing followed by chromizing sequence yields a duplex coating with superior wear and corrosion resistance compared to other sequences. Based on this, the two-step pack cementation process of pre-boronizing followed by chromizing was adopted in this study to prepare boron-chromium composite coatings.

To the best of our knowledge, no systematic research has been reported on the friction-wear and high-temperature oxidation properties of borochromized coatings on the surface of nickel-based alloys to date. It remains unclear whether borochromized coatings can enhance the high-temperature oxidation resistance while retaining the excellent friction-wear performance of single boronized coatings, nor has the specific improvement extent and intrinsic action mechanism of such composite coatings on the high-temperature oxidation resistance of single boronized coatings been elucidated. Therefore, this study aims to evaluate the friction-wear performance and high-temperature oxidation resistance of borochromized coatings. By comparing the properties with those of single boronized coatings, single chromized coatings and the substrate alloy, the wear mechanism and high-temperature oxidation protection mechanism of borochromized coatings are clarified.

## 2. Materials and Methods

The primary chemical composition of the GH3039 nickel-based alloy employed in this study is presented in Table 1. Prior to boronizing, chromizing, and borochromizing treatments, GH3039 alloy rods were cut into sheet samples with dimensions of Φ25 mm × 2 mm using wire-electrode cutting. The samples were ground using SiC sandpapers from 80 to 1200 grit, followed by ultrasonic cleaning in ethanol for 5 min to remove contaminants.

The processed samples were embedded in a boronizing media (90 wt% B_4_C, 10 wt% KBF_4_) and placed in an alumina crucible. The crucible was then sealed with refractory clay and dried at 120 °C for 2 h to remove moisture. Boronizing was performed in a muffle furnace at 950 °C for 8 h. After treatment, the samples were removed from the furnace, cooled, and ultrasonically cleaned in anhydrous ethanol to eliminate adherent boronizing residues.

For chromizing treatment, the samples were embedded in a chromizing media (50 wt% Cr, 46 wt% Al_2_O_3_, 4 wt% NH_4_Cl) and preheated at 70 °C for 6 h to activate the medium. They were then transferred to a tube furnace under an argon protective atmosphere and heated for subsequent chromizing. Chromized samples were isothermally held at 1100 °C for 9 h, whereas borochromized samples were held at 950 °C for 9 h. After treatment, all samples were ultrasonically cleaned in anhydrous ethanol for 30 min to remove residual chromizing medium. The surface-treated samples were subjected to cutting, cross-section mounting, grinding (80 to 2000 grit SiC paper) and mechanical polishing.

The microstructure and cross-sectional morphology of the coatings were characterized via a scanning electron microscopy (SEM, FEI Sirion 200, Field Electron and Ion Company, Hillsboro, OR, USA). Elemental distribution was analyzed via energy-dispersive X-ray spectroscopy (EDS, with the built-in detector of the SEM, Field Electron and Ion Company, Hillsboro, OR, USA) and electron probe microanalysis (EPMA, JEOL JXA-8230, Japan Electron Optics Laboratory, Ltd., Tokyo, Japan). Phase composition of the coating surface was determined by X-ray diffraction (XRD, Rigaku D/Max 2500, Rigaku Corporation, Tokyo, Japan), with Cu Kα radiation (λ_Cu_ = 0.1540 nm) over a 2*θ* range of 5–90° at a scanning rate of 5.0°/min.

High-temperature oxidation resistance was evaluated via a static oxidation test. As-received GH3039 alloy, boronized, chromized, and borochromized samples were oxidized in a muffle furnace at 850 °C and 950 °C for a total of 100 h. Samples were retrieved at 10 h intervals to measure changes in dimensional changes and mass gain.

Vickers microhardness of the coating surfaces and cross sections was measured using a Vickers hardness tester (Huayin 200HBVS-30, Laizhou Huayin Testing Instrument Co., Ltd., Laizhou, China). Ten replicate measurements per sample were taken, and the average values were reported. The tests were performed under loads of 50 gf, respectively, with a dwell time of 15 s. Room-temperature tribological behavior was evaluated using an SRV-4 reciprocating friction and wear tester (Optimol Instruments Prüftechnik GmbH, Munich, Germany), with Si_3_N_4_ balls (6 mm in diameter) as the counterbodies. The test parameters were set as follows: applied load of 50 N, stroke length of 10 mm, sliding speed of 66.6 mm/s, and test duration of 30 min. The coefficient of friction (CoF) was automatically recorded, with three replicate tests per each sample to obtain the average CoF. Wear tracks were calculated via a Wyko NT9100 white light interference profilometer (Veeco Instruments Inc., Tarrytown, NY, USA), and wear volume was calculated via profilometric analysis. The final wear volume value for each sample was the average of three replicate measurements.

## 3. Results and Discussion

### 3.1. Microstructure and Phase Analysis

**Figure 1:**
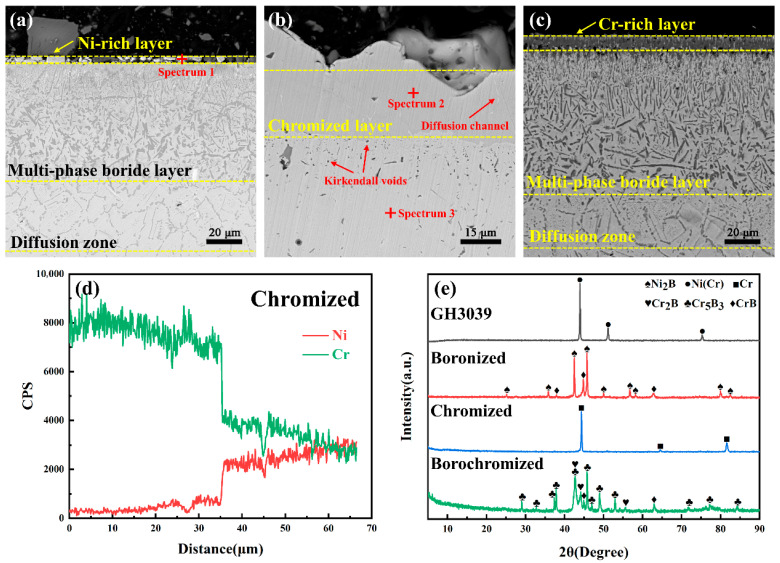
Cross-sectional SEM images of the coatings on GH3039 alloy: (**a**) boronized coating; (**b**) chromized coating; (**c**) borochromized coating; (**d**) line scan results of chromized coating; (**e**) XRD analysis of GH3039 alloy and coatings.

Figure 1 shows the cross-sectional morphologies of boronized, chromized, and borochromized coatings. As shown in Figure 1a, the outermost region of the boronized coating is a 3.6 μm-thick Ni-rich layer, which is prone to spalling. The Ni content at spectrum 1 reaches 97.2% (EDS results in Table 2). To ensure reliable subsequent performance testing, this layer was carefully polished off prior to analysis. The intermediate layer is a 74.8 μm-thick multiphase boride layer, exhibiting a dense spot-like structure in its upper region and a dendritic grain morphology in the lower region. The innermost layer is a 36.5 μm-thick boron diffusion layer with a coarse mesh-like structure. Figure 1b shows the cross-sectional morphology of the chromized coating, revealing a bilayer structure. The surface layer is a continuous, dense Cr-rich layer (32.6 μm thick) with excellent adhesion to the substrate. EDS analysis of spectrum 2 and 3 reveals a significantly higher Cr content in this layer compared to the substrate. Within the coating, fine outward diffusion channels of Ni are observed, and distinct Kirkendall voids resulting from interdiffusion are present at the coating-substrate interface. The sublayer is a 25.6 μm-thick chromium diffusion layer, where the Cr content also exceeds that of the substrate.

Figure 1c shows the cross-sectional morphology of the borochromized coating. Subjected to two thermal diffusion treatments, this coating is significantly thicker than the single boronized coating and comprises three distinct regions: an outermost 22.8 μm-thick Cr-rich layer, an intermediate 72.8 μm-thick multiphase boride layer, and an innermost 40.8 μm-thick boron diffusion layer. Notably, the increase in the overall coating thickness is mainly attributable to the formation of the surface Cr-rich layer, whereas the thickness of the multiphase boride layer remains nearly unchanged. The microstructure of the multiphase boride layer in the borochromized coating is similar to that of the single boronized coating, with a dense spot-like structure in the upper region and a dendritic grain morphology in the lower region.

Figure 1e shows the XRD phase analysis results for all samples. GH3039 is a typical solid-solution strengthened nickel-based alloy. Since the atomic radius of Cr is slightly larger than that of Ni, the substitution of Ni atoms with Cr in the solid solution induces lattice expansion, which shifts XRD diffraction peaks to lower angles [20,21]. For the boronized coating, the dominant surface phases are Ni_2_B and CrB. The chromized coating is primarily composed of α-Cr, as the chromium content exceeds its solid solubility limit in GH3039 during treatment. On the borochromized coating surface, no Ni_2_B phase is detected due to the enrichment of Cr and B; instead, the surface is predominantly composed of CrB, Cr_2_B and Cr_5_B_3_.

**Figure 2:**
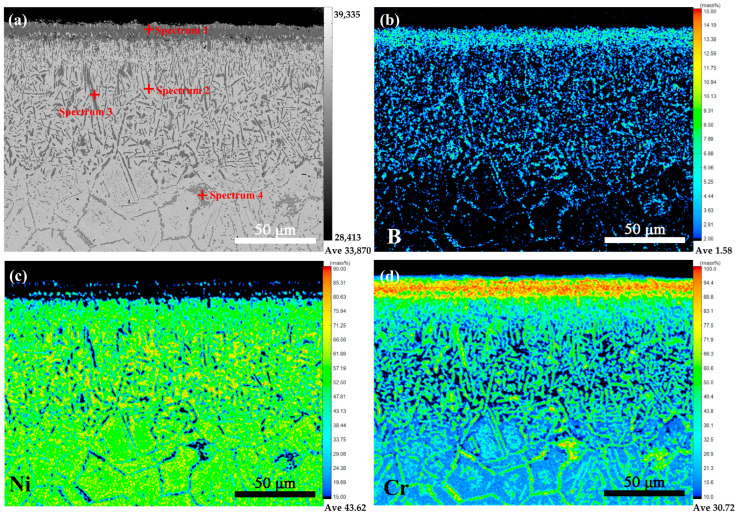
EPMA images of the borochromized sample: (**a**) cross-sectional morphology; (**b**) B distribution; (**c**) Ni distribution; (**d**) Cr distribution.

To further elucidate the elemental distribution characteristics of the borochromized coating, EPMA analysis was conducted. Figure 2 shows the EPMA results for the borochromized coating. As shown in Figure 2b, in the Cr-rich layer, obvious enrichment of Cr and B is observed, with Ni nearly absent. Boron atoms are widely distributed throughout the coating in spot-like, dendritic, and dispersed morphologies, indicating that boron is predominantly concentrated in boride phases rather than undergoing uniformly dissolution in the substrate. EPMA point-scan results (Table 3) reveal additional details: data for spectrum 2 and 3 reveal that the dendritic grain regions within the multiphase boride layer are enriched in Cr and B, whereas the adjacent regions exhibit Ni enrichment. This phenomenon is attributed to the more negative mixing enthalpy (ΔHm) of Cr–B atomic pairs compared with that of Ni-B pairs at low boron concentration, favoring the formation of chromium borides. During the high-temperature secondary chromizing process, boron atoms underwent redistribution and were enriched in the Cr-rich layer, which is also consistent with the characteristic that the lower mixing enthalpy of Cr–B promotes the formation of chromium borides. Wu et al. [12] confirmed the dendritic phase as Cr_2_B via transmission electron microscopy (TEM). The bottom diffusion layer is formed by the rapid diffusion of B atoms [22], with boron atoms primarily diffusing along grain boundaries. EPMA results at spectrum 4 verify the elevated B content at these boundaries.

### 3.2. Hardness and Wear Performance

**Figure 3:**
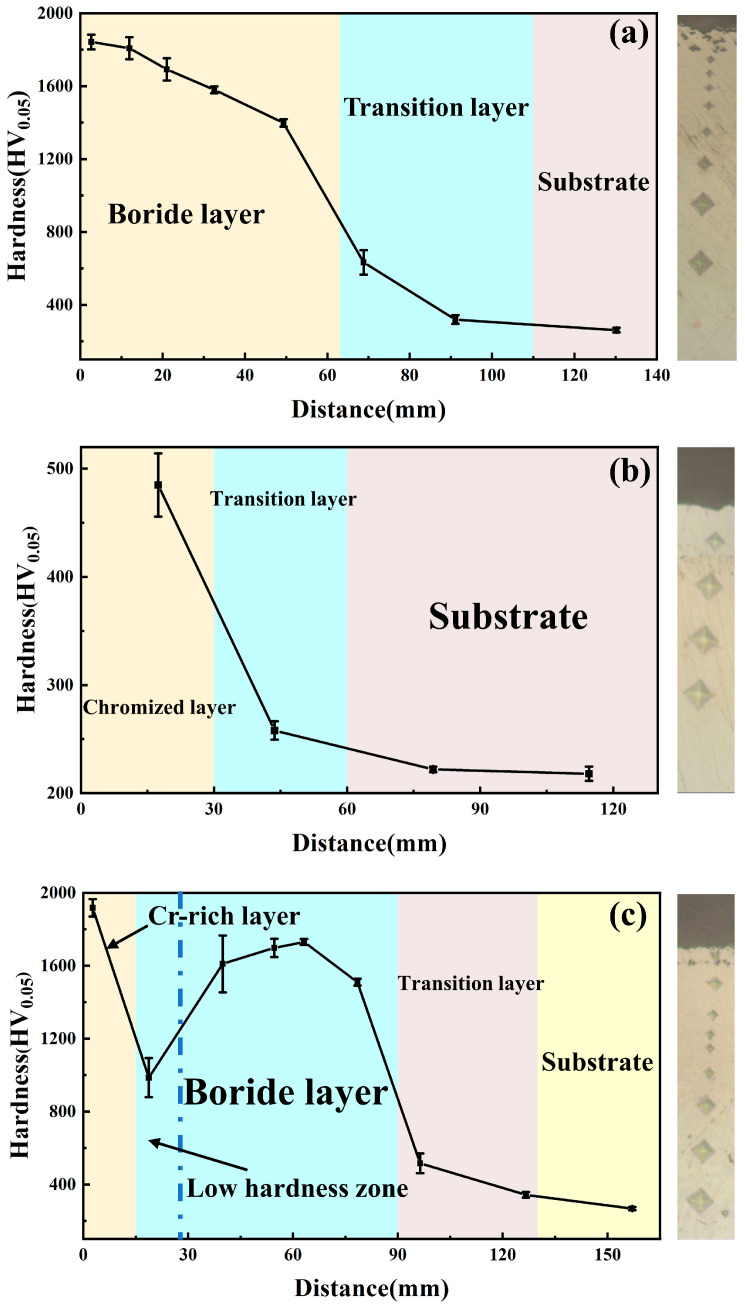
Hardness gradient of samples: (**a**) boronized; (**b**) chromized; (**c**) borochromized.

Figure 3 presents the Vickers hardness profiles across the cross sections of the boronized, chromized, and borochromized coatings. After boronizing, the boronized coating achieves a maximum hardness of 1843 HV_0.05_. Chromizing slightly increases the surface hardness, with the chromized coating reaching a maximum hardness of 485 HV_0.05_. The borochromized coating exhibits the highest hardness among all samples, with a maximum value of 1918 HV_0.05_. The coating hardness gradually decreases with increasing distance from the surface, displaying a gradient distribution. In the borochromized coating, the Cr-rich layer shows the highest hardness owing to the enrichment of chromium boride phases. However, a distinct hardness-softening zone is observed at the interface between the Cr-rich layer and the multiphase boride layer. Combined with the preceding EPMA results and cross-sectional morphological analysis, the formation of this softening zone can be attributed to the synergistic effect of multiple factors. During the high-temperature secondary chromizing treatment following the initial boronizing process, because Cr–B atomic pairs possess a more negative enthalpy of mixing, B atoms in the pre-formed boronized layer tend to migrate toward the Cr-rich layer and undergo redistribution. As a result, the B content near the interface decreases markedly, leading to a reduced volume fraction of hard second phases and consequently weakening the continuity of the boride layer. Meanwhile, in this multi-element diffusion system, the difference in diffusion rates between the inward diffusion of Cr and the counter-diffusion of Ni and other elements gives rise to vacancy accumulation, which further results in the formation of Kirkendall voids at the interface. Although these voids are not the fundamental cause of the hardness reduction, they impair the local load-bearing capacity of the interfacial region and tend to enlarge the indentation during hardness testing, thereby further aggravating the apparent hardness softening. The presence of Kirkendall voids has been confirmed by the SEM image in Figure 1c. Therefore, this interfacial region exhibits relatively low local hardness. The pronounced hardness gradient between the high-hardness Cr-rich layer and the interfacial softening zone may adversely affect the tribological performance of the coating, which should be further verified by subsequent wear tests.

**Figure 4:**
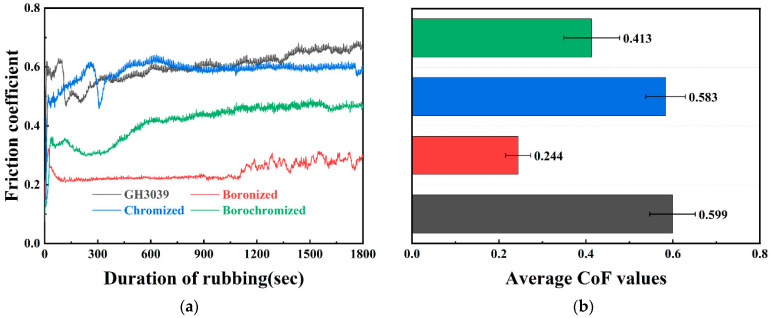
Room-temperature friction behavior of GH3039 alloy, boronized, chromized, and borochromized samples: (**a**) COF curve at 50 N; (**b**) average COF at 50 N.

Friction and wear tests were conducted on as-received, boronized, chromized, and borochromized GH3039 samples. Figure 4 shows the CoF versus time curves and the corresponding average CoF values for all samples under a 50 N load at room temperature. The CoF-time profiles reveal that the CoF first fluctuates during the initial wear stage and then stabilizes in the subsequent steady-state wear stage. At 50 N, the average CoF values for the as-received GH3039, boronized, chromized, and borochromized samples were 0.599, 0.244, 0.583, and 0.413, respectively. Both the boronized and borochromized specimens exhibited lower CoF than the as-received GH3039 alloy, with the boronized specimens showing the lowest value. In contrast, the CoF of the chromized samples was nearly identical to that of the as-received GH3039 alloy.

The reduced in the friction coefficient primarily arises because both boronizing and borochromizing treatments form a boride layer of ultrahigh hardness on the substrate surface. This layer mitigates plastic deformation during contact, thereby reducing frictional forces. Furthermore, according to research by Erdemir et al. [23], during wear, the boride phase (e.g., CrB, Ni_2_B) in the coating reacts with O to form boron oxide (B_2_O_3_). The relevant reactions areas follows:

(1) $$2\mathrm{CrB}\;+\;3O_{2(g)}\;↔\;{\mathrm{Cr}}_{2}O_{3}\;+\;B_{2}O_{3}$$

(2) $$2{\mathrm{Ni}}_{2}B+3.5O_{2}\;↔\;4\mathrm{NiO}\;+\;B_{2}O_{3}$$

The B_2_O_3_ films act as solid lubricant, effectively lowering the friction coefficient of the specimens. In conclusion, boronizing and borochromizing treatments significantly reduce the friction coefficient of the GH3039 alloy, thus enhancing its tribological performance.

**Figure 5:**
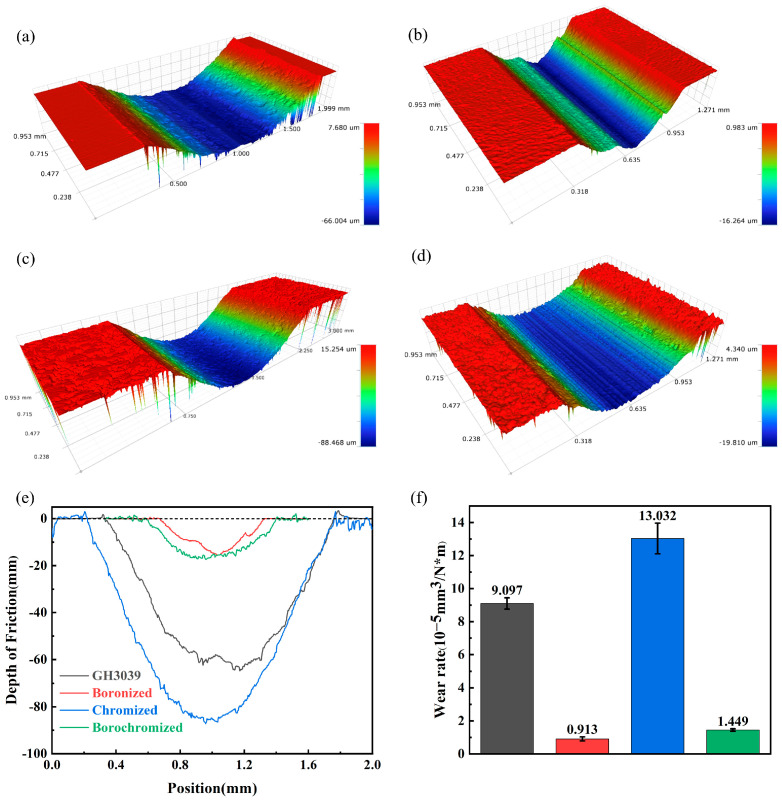
Three-dimensional wear morphology of GH3039 alloy and coating under 50 N load: (**a**) GH3039; (**b**) boronized; (**c**) chromized; (**d**) borochromized; (**e**) wear depth under 50 N load; (**f**) wear rate under 50 N load (The dashed lines represent the surfaces of the samples).

Figure 5 shows the three-dimensional wear morphologies, wear depths and wear rates of all tested specimens under a 50 N applied load. In friction and wear testing, the wear rate serves as a key parameter for evaluating material wear resistance. The specific volume wear rate *K* is calculated using the following equation:

(3) $$K\;=\;\frac{V}{F\;\times \;d}$$

where *V* denotes the worn volume loss, *F* is the applied load, and *d* represents the total sliding distance of the sample [24,25]. Under a 50 N load, the as-received GH3039 alloy exhibits a wear depth of 64.67 µm and a wear rate of 9.09 × 10^−5^ mm^3^·N^−1^·m^−1^. Following boronizing treatment, the boronized sample showed a significantly reduced wear rate of 0.91 × 10^−5^ mm^3^·N^−1^·m^−1^, which is a decrease of approximately one order of magnitude compared to the substrate. In contrast, the chromized sample exhibited the maximum wear depth and width, indicating severe wear and poor wear resistance, with a relatively high wear rate of 13.03 × 10^−5^ mm^3^·N^−1^·m^−1^. For the borochromized sample, uniform wear was observed, with a wear rate of 1.44 × 10^−5^ mm^3^·N^−1^·m^−1^, representing an 84.07% reduction relative to the substrate. Thus, borochromizing treatment effectively enhances the tribological performance of the GH3039 alloy, as evidenced by its superior wear resistance compared to both the as-received substrate.

**Figure 6:**
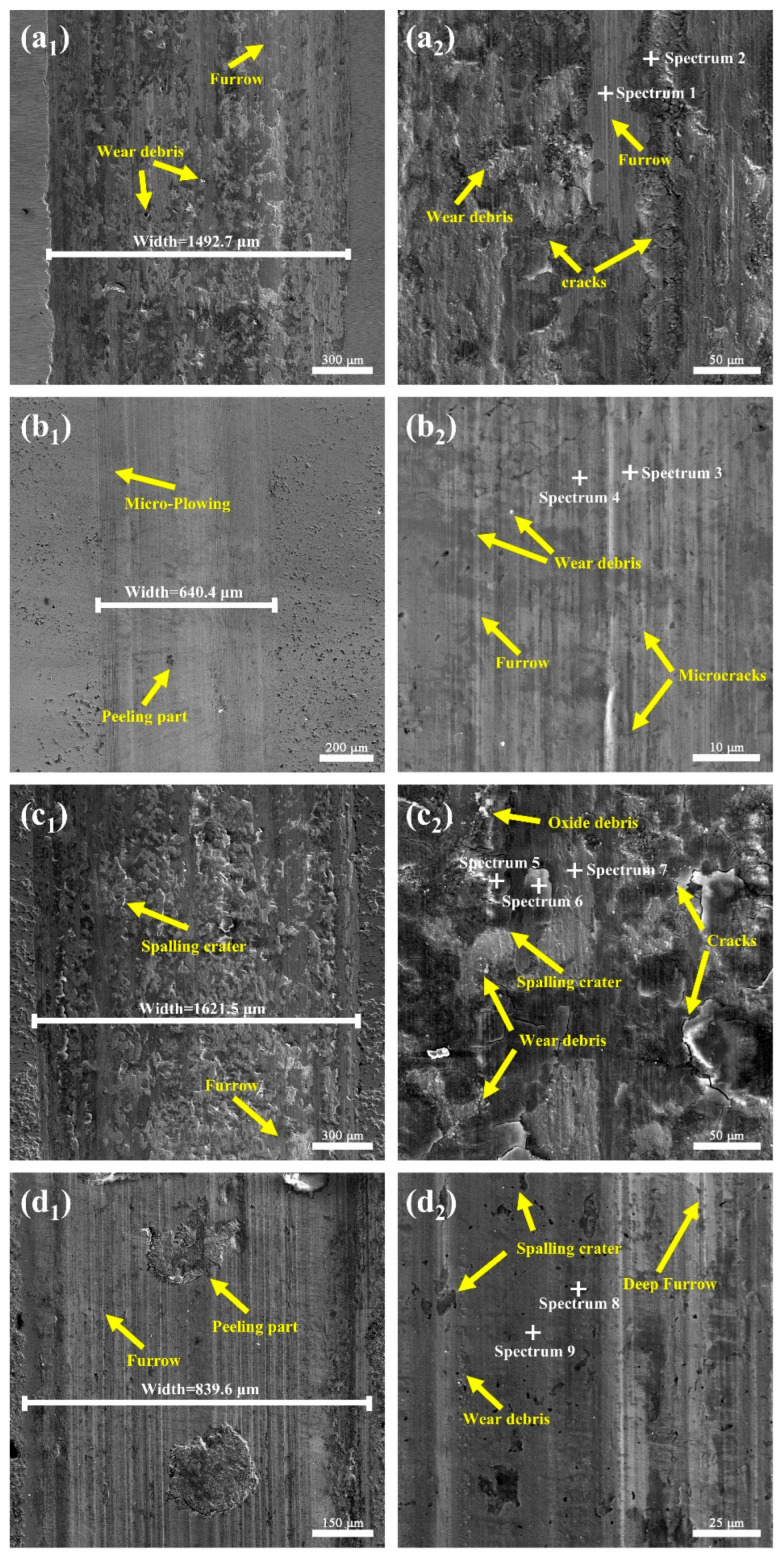
SEM images of the wear surfaces of GH3039 and the coatings under 50 N load: (**a_1_**,**a_2_**) GH3039; (**b_1_**,**b_2_**) boronized coating; (**c_1_**,**c_2_**) chromized coating; (**d_1_**,**d_2_**) borochromized coating.

To further elucidate the wear characteristics of each sample, SEM and EDS analyses were performed. The EDS results are shown in Table 4, and Figure 6 shows the SEM micrographs of all samples after 30 min of wear under a 50 N load. As shown in Figure 6a_1_,a_2_, the as-received GH3039 alloy exhibits severe wear, with a wear track width of 1492.7 µm. The worn surface is characterized by numerous long, deep, continuous scratches, grooves and extensive spalling. EDS analysis at Points 1 and 2 detected Si, indicating significant elemental transfer during friction—a hallmark of adhesive wear. Additionally, a high concentration of O was detected at Point 2, revealing severe oxidative wear. During friction, generated frictional heat accelerates the reaction between the metal surface and atmospheric O_2_, forming an oxide layer. Sustained friction causes spallation of this layer, exposing fresh metal surface that re-oxidize cyclically. This repeated oxidation-spalling process further accelerates the wear rate [26,27,28]. Therefore, the wear mechanism of the as-received GH3039 alloy involves a synergistic effect of adhesive wear (elemental transfer), oxidative wear (cyclic oxidation-spallation), and fatigue wear (from crack propagation induced by repeated loading).

As shown in Figure 6b_1_,b_2_, the boronized specimen exhibits distinct surface scratches and microcracks. However, unlike the substrate, minimal spallation pits or residual wear debris was observed. Abrasive wear dominates its wear mechanism. This behavior stems from the boride layer formed during boronizing, which significantly increases the surface hardness. The enhanced hardness mitigates local material spallation and reduces the penetration depth of abrasive particles. It also suppresses the propagation length and depth of microcracks. Furthermore, the low friction coefficient of the boronized specimen minimizes frictional heat accumulation, thereby reducing fatigue wear induced by thermal stress [29,30,31,32].

The chromized specimen exhibits the most severe wear behavior. Since its wear depth far exceeds the thickness of the chromized coating, wear propagates into the substrate, resulting in a wear morphology similar to that of the GH3039 alloy but considerably more severe. The worn surface is characterized by numerous cracks and spallation, with abundant wear debris and oxide particles remaining. EDS analysis at Points 5 and 6 revealed a high Si content, confirming severe adhesive wear. Although chromizing treatment marginally increases surface hardness, it also elevates surface brittleness, rendering the specimen susceptible to crack propagation and spallation under friction. In addition, the mismatch in thermal expansion coefficients between the coating and substrate leads to internal stress accumulation, ultimately causing coating cracking or spallation during friction [33,34]. Thus, the chromized specimen exhibits an accelerated wear rate, ultimately resulting in inferior wear resistance compared to the as-received GH3039 alloy.

The worn surface of the borochromized specimen is presented in Figure 6d_1_,d_2_. Low-magnification SEM images show deep grooves and two large spallation pits after wear under a 50 N load, attributed to poor load-bearing capacity and a large hardness gradient of the hardness-softening zone. The presence of wear debris and microvoids suggests that abrasive particles embed themselves into the surface, physical interlocking with the substrate, and mechanically abrade it, ultimately leading to void formation. At higher magnification (Figure 6d_2_), the relatively high surface hardness results in a uniform, smooth worn surface with no flaking or delamination typically observed in the GH3039 alloy. This confirms that borochromizing treatment significantly enhances the tribological performance of the specimen.

**Figure 7:**
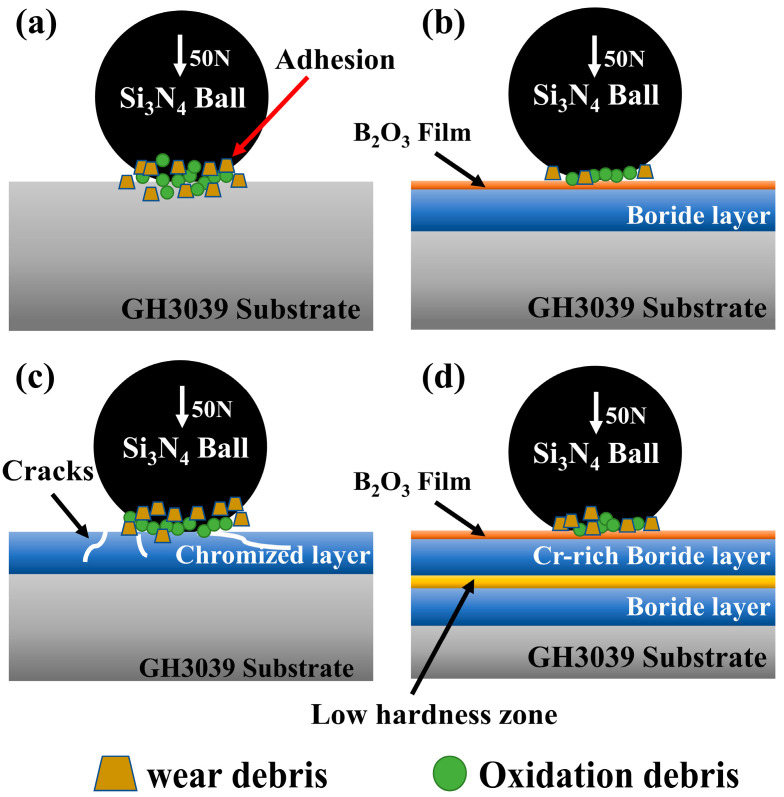
Schematic diagram of the wear mechanisms of GH3039 alloy and coatings under 50 N load: (**a**) GH3039; (**b**) boronized coating; (**c**) chromized coating; (**d**) borochromized coating.

Figure 7 summarizes the wear mechanisms of all samples under a load of 50 N, with the wear behaviors and mechanisms of the samples exhibiting distinct differences. The GH3039 alloy exhibited prominent adhesive wear characteristics. During the wear process, intense frictional heat was generated at the contact points between the Si_3_N_4_ counterbody and the GH3039 surface, inducing a sharp rise in local temperature and subsequent thermoplastic deformation even with small-area melting. This resulted in severe adhesion on the substrate surface, with material adhering to the Si_3_N_4_ ball. As wear proceeded, the temperature at the contact points increased further, causing the interfacial adhesion to become unstable; the adhered substrate material then spalled off the surface, forming numerous spalling craters. The wear debris generated by spalling and oxide debris formed during friction were continuously pressed into the contact interface between the Si_3_N_4_ ball and the substrate, acting as hard abrasive particles and exacerbating interfacial mechanical friction. This not only drastically increased the friction force and wear rate but also triggered a severe coupling behavior of adhesive wear and abrasive wear, ultimately leading to severe wear of the substrate [35,36].

The wear of the boronized and borochromized samples was dominated by abrasive wear. The high-hardness boride layer formed on the surface could effectively disperse contact stress and significantly suppress thermoplastic deformation and melting of the contact surface, thus avoiding the plastic flow and interfacial adhesion observed in the substrate. Meanwhile, the B_2_O_3_ film formed during the friction and wear process could effectively isolate the direct contact between the coating and the counterbody, drastically reducing the friction coefficient and the accumulation of interfacial frictional heat, and fundamentally inhibiting the occurrence of adhesive wear [37]. In addition, the high-hardness hardened layer could effectively resist the cutting and scratching of hard abrasive particles, significantly reducing surface damage caused by abrasive wear. However, the presence of a hardness-softening zone between the Cr-rich layer and the multiphase boride layer in the borochromized coating, along with a large hardness gradient between this zone and the Cr-rich layer, led to the formation of a small number of large spalling craters on the borochromized sample during wear, ultimately resulting in its wear resistance being slightly inferior to that of the boronized coating.

The wear resistance of the chromized sample was worse than that of the substrate. Although the surface coating slightly increased the hardness, it significantly enhanced the brittleness of the material. Under frictional stress, microcracks were prone to rapid initiation and propagation inside the coating, eventually leading to localized brittle spalling of the coating. After the coating spalled off, the substrate was directly exposed to the counterbody, undergoing severe adhesive wear consistent with the substrate. Furthermore, the spalled coating debris acted as hard abrasive particles to aggravate wear, ultimately resulting in severe wear behavior coupled with adhesive wear and brittle spalling.

### 3.3. High-Temperature Oxidation Resistance

**Figure 8:**
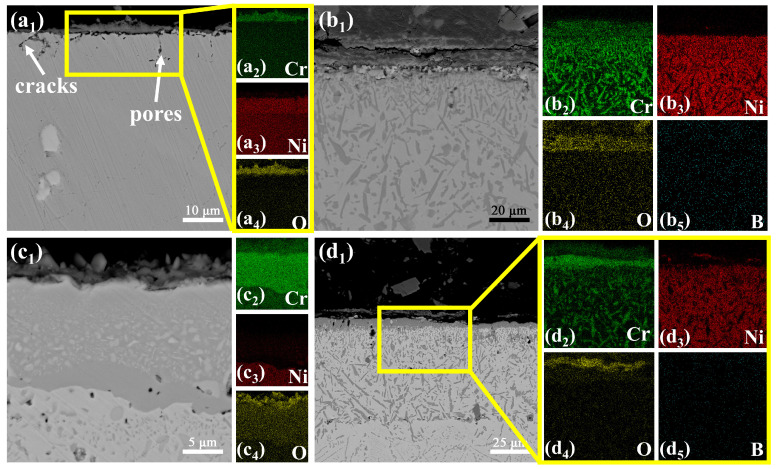
Cross-sectional morphologies of specimens after 100 h oxidation at 850 °C: (**a_1_**–**a_4_**) GH3039 alloy; (**b_1_**–**b_5_**) boronized coating; (**c_1_**–**c_4_**) chromized coating; (**d_1_**–**d_5_**) borochromized coating.

**Figure 9:**
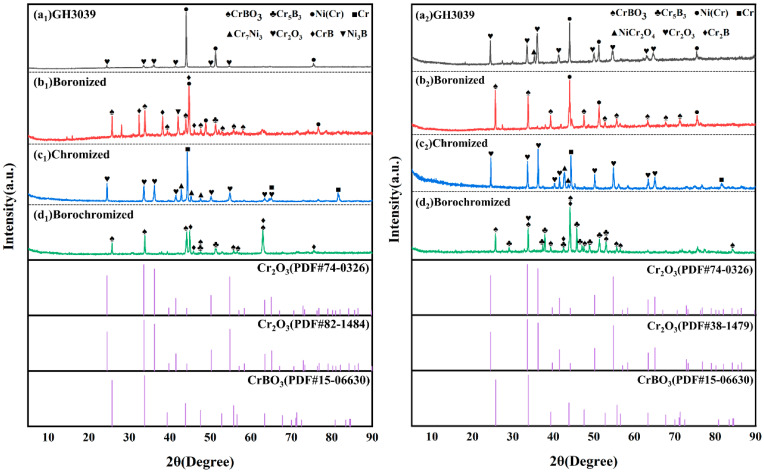
XRD patterns of samples after 100 h of oxidation: (**a_1_**–**d_1_**) at 850 °C; (**a_2_**–**d_2_**) at 950 °C.

To investigate the high-temperature oxidation resistance of as-received GH3039 alloy, and boronized, chromized and borochromized specimens, static oxidation tests were conducted at 850 °C and 950 °C. Figure 8 presents the SEM images of the cross-sectional morphology of each specimen after 100 hours of oxidation at 850°C, and Figure 9 shows the XRD results of the specimens after 100 hours of oxidation at 850°C and 950°C. Figure 8a_1_ reveals that after 100 h of exposure at 850 °C, the as-received GH3039 alloy underwent mild oxidation, forming an oxide layer of non-uniform thickness and an average thickness of 2.98 μm. Elemental mapping (Figure 8a_2_–a_4_) further demonstrates enrichment of Cr and O within the oxide layer, and XRD analysis confirms that the layer is primarily composed of Cr_2_O_3_. Fine cracks are observed within the oxide layer, and pores are present at the oxide layer-substrate interface. A small portion of these pores propagate into cracks, leading to poor adhesion between the oxide layer and the substrate. Additionally, cracks and pores are detected on the substrate surface beneath the oxide layer. These defects facilitate O infiltration into the substrate interior, accelerate O diffusion, and thus exacerbate substrate oxidation [38,39,40,41]. Consequently, owing to the porous oxide structure and weak oxide-substrate bonding, the protective effect of the oxide layer on the substrate is limited.

Figure 8b_1_–b_5_ show that after 100 h oxidation at 850 °C, the boronized specimen formed a relatively thick oxide layer with an average thickness of 5.04 μm. Minor cracks and local spallation were observed within the oxide layer, and a gap existed at the oxide layer-coating interface. Despite these surface defects, the coating beneath the oxide layer remained intact and dense. The boride layer thinned slightly compared to its pre-oxidation state, while the diffusion layer thickened significantly. XRD analysis verifies that the oxide layer was primarily composed of CrBO_3_, with O enriched exclusively on the coating surface, indicating excellent protective performance at this temperature. This is attributed to the self-healing capability of B_2_O_3_, a byproduct of boron oxidation, B_2_O_3_ fills microcracks in the coating and reduces O penetration until its volatilization at higher temperatures. Thus, boronizing treatment effectively enhances the coating’s high-temperature oxidation resistance. Figure 8c_1_ presents the cross-sectional morphology of the chromized specimen after 100 h oxidation at 850 °C. Mild oxidation occurred on the coating surface, forming an oxide layer with an average thickness of 2.22 μm and XRD analysis revealed a Cr_2_O_3_ oxide layer with unoxidized α-Cr still present. The chromized layer retained dense, enabling effective protection of the substrate and significantly improving the alloy’s high-temperature oxidation resistance. Figure 8d_1_ displays the cross-sectional morphology of the borochromized specimen after 100 h oxidation at 850 °C, showing a thin oxide layer with an average thickness of 2.68 μm. Elemental mapping (Figure 8d_2_–d_5_) shows the Cr-rich layer remained intact, with O confined to the outermost surface, demonstrating effective inhibition of O diffusion. XRD results indicated that the oxide layer was primarily composed of CrBO_3_, with a large amount of Cr_5_B_3_ and CrB phases also detected. The multiphase boride layer retained a morphology almost identical to its pre-oxidation state, confirming structural preservation. In conclusion, boronizing, chromizing and borochromizing treatments all remarkably enhance the high-temperature oxidation resistance of GH3039 alloy at 850 °C.

**Figure 10:**
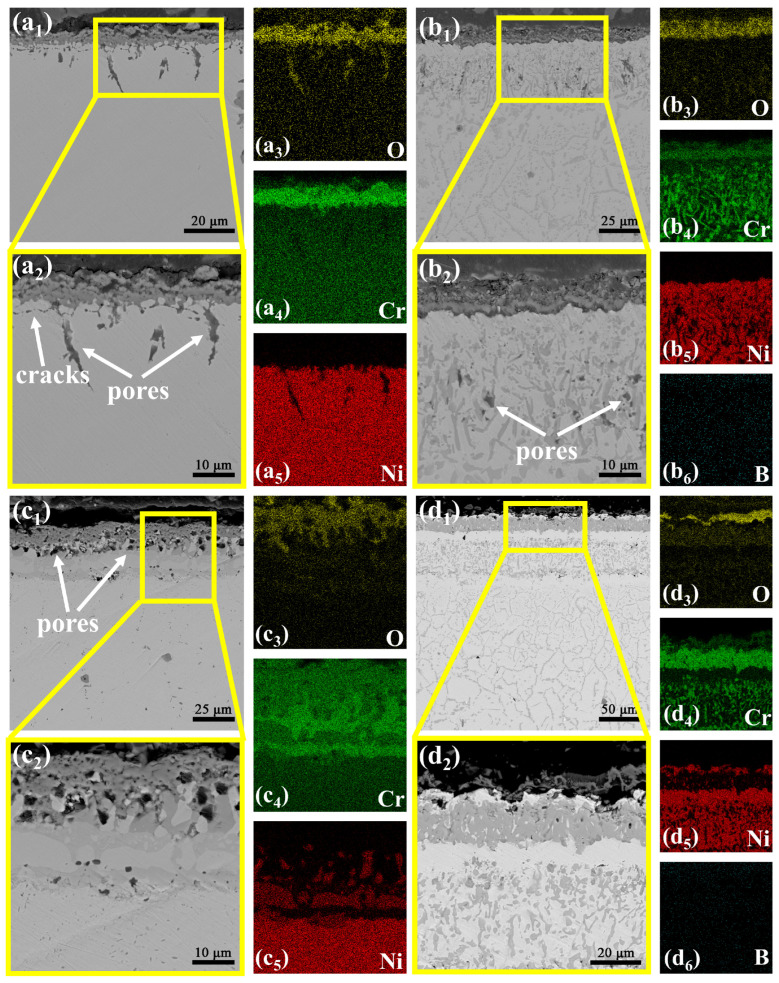
Cross-sectional morphologies of specimens after 100 h oxidation at 950 °C: (**a_1_**–**a_5_**) GH3039 alloy; (**b_1_**–**b_6_**) boronized coating; (**c_1_**–**c_5_**) chromized coating; (**d_1_**–**d_6_**) borochromized coating.

Figure 10 presents the cross-sectional morphologies of as-received GH3039 alloy, boronized, chromized and borochromized specimens after 100 h of oxidation at 950 °C. At this temperature, the oxidation behavior of all specimens became considerably more pronounced. Figure 10a_1_ shows the cross-sectional morphology of as-received GH3039 alloy after 100 h oxidation at 950 °C. The alloy underwent severe oxidation, forming a relatively thick oxide layer on the surface with an average thickness of 7.28 μm. Elemental mapping (Figure 10a_2_–a_5_) reveals distinct enrichment of Cr and O on the surface. Combined with XRD analysis, the oxide layer is confirmed to be primarily composed of Cr_2_O_3_. While Cr_2_O_3_ films typically inhibit further O diffusion [42,43,44], the oxide layer on the GH3039 alloy exhibits obvious pores and cracks. Pronounced pores and cracks are also observed on the substrate beneath the oxide layer, and these defects exacerbate internal oxidation of the substrate. Figure 10a_3_ further demonstrates that O has diffused deep into the substrate along these pores and cracks, indicating that the Cr_2_O_3_ film can no longer effectively impede O diffusion at 950 °C. Consequently, the GH3039 alloy exhibits significant oxidation weight gain and poor high-temperature oxidation resistance at this temperature. As shown in Figure 10b_1_,b_2_, the oxide layer on the boronized specimen features a loose structure with numerous pores and an average thickness of 8.56 μm. The dot-like regions at the top of the boronized coating have disappeared, and the coating thickness was drastically reduced from 74.8 μm (pre-oxidation) to 27.1 μm (post-oxidation). A large number of cracks and pores formed within the coating, and the diffusion layer thickened significantly, indicating damage to the boronized coating’s microstructure. Mechanistically, nickel borides and chromium borides dispersed in the coating react with O at high temperatures to form NiO, Cr_2_O_3_ and B_2_O_3_. Volume changes during the oxidative degradation of these borides induce internal cracking. A portion of B_2_O_3_ reacts with Cr_2_O_3_ to form CrBO_3_, while the remaining B_2_O_3_ volatilizes at high temperatures due to its low melting point. The volatilization of B_2_O_3_ not only induces cracks and pores but also triggers coating cracking and spallation, impairing the durability of the oxide film [45]. Owing to the volatility of B_2_O_3_, it is not detected by XRD analysis, and only trace amounts of O are detected in the substrate interior.

Figure 10b_3_–b_6_ shows that Ni is enriched beneath the oxide layer, while Cr is depleted in this region. This depletion results in an insufficient Cr supply for the continuous formation of Cr_2_O_3_ films during subsequent oxidation. Figure 10b_3_ indicates that O has not diffused into the substrate, suggesting that the boronized coating provides partial protective to the substrate. However, the damaged microstructure, increased porosity, and drastic thinning of the boronized coating lead to a remarkable decline in its protective performance at high temperatures. In conclusion, although the boronized coating exhibits a temporary barrier effect in the early oxidation stage, its poor thermal stability at elevated temperatures renders it unable to provide effective long-term protection for the substrate.

Figure 10c_1_,c_2_ displays the cross-sectional morphology of the chromized specimen after 100 h oxidation at 950 °C, revealing a relatively thick, loose, and porous oxide layer on the surface with an average thickness of 10.76 μm. As shown in Figure 10c_3_–c_5_, the chromized layer does not fail completely, but its thickness significantly reduces from 32.6 μm (pre-oxidation) to 12.4 μm (post-oxidation). Meanwhile, Cr distribution in the layer becomes inhomogeneous with a decreased Cr content, and a large amount of Ni diffuses outward. Mechanistically, the difference in diffusion coefficients between Ni and Cr induces pore formation within the chromized layer. XRD analysis confirms the surface oxide layer is mainly composed of NiCr_2_O_4_ and Cr_2_O_3_. The mismatch in thermal expansion coefficients among Cr_2_O_3_ and NiCr_2_O_4_ further increases interfacial stress between the oxide layer and chromized layer, promoting pores and cracks formation [46,47,48]. In conclusion, chromizing treatment provides partial oxidation protection for the GH3039 alloy. However, the significant thinning and high porosity of the coating after oxidation indicate poor oxidation resistance under prolonged high-temperature conditions.

Figure 10d_1_,d_2_ shows the cross-sectional morphologies of the borochromized specimen after oxidation at 950 °C for 100 h. It can be seen that only a thin oxide layer with an average thickness of approximately 6.84 μm formed on the specimen surface. Combined with the XRD analysis, the oxide products were mainly composed of Cr_2_O_3_ and CrBO_3_. After oxidation, the overall coating structure remained intact, and the morphologies of the Cr-rich layer and the multiphase boride layer were basically consistent with those before oxidation, while the thickness of the Cr-rich layer changed only slightly. Meanwhile, during prolonged oxidation, B continued to diffuse inward, resulting in a significant thickening of the bottom diffusion layer, whereas the thickness of the multiphase boride layer decreased slightly from 72.8 μm to 63.9 μm. As shown in Figure 10d_3_–d_6_, O was mainly enriched in the outermost region, indicating that the dense Cr-rich layer effectively hindered the inward diffusion of oxygen into the coating. Obvious Ni enrichment was observed beneath the Cr-rich layer, whereas Ni was hardly detected within the Cr-rich layer, suggesting that this layer exerted a marked inhibitory effect on the outward diffusion of Ni. Compared with the α-Cr layer in the chromized coating, the continuous and dense chromium-boride-enriched layer, owing to its lower Ni solubility and higher diffusion barrier, can retard the interdiffusion of Cr and Ni at the interface to a certain extent, thereby reducing the tendency for Kirkendall void formation and improving the structural stability of the coating. At the same time, compared with the dispersed chromium borides in the boronized coating, the continuous and dense Cr-rich layer formed on the borochromized specimen is more favorable for the formation of a stable and compact oxide film, thus providing more effective protection for the substrate.

In addition, the high Cr content in the Cr-rich layer provides a sufficient Cr source for the rapid formation of a Cr_2_O_3_ film during oxidation [49,50]. The Cr_2_O_3_ film formed at the early oxidation stage not only effectively suppresses the further oxidation of the borides, but also promotes the conversion of part of the boron oxidation products into relatively stable CrBO_3_, thereby reducing the volatilization tendency of B_2_O_3_ and improving the compactness and stability of the oxide film. In summary, the borochromizing treatment significantly enhances the high-temperature oxidation resistance of the GH3039 alloy at 950 °C.

**Figure 11:**
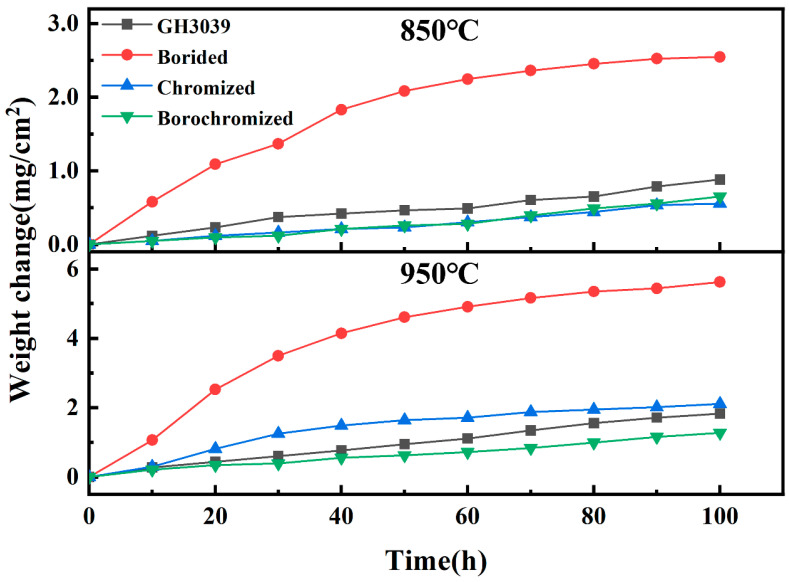
Weight gain curves of the substrate, boronized, chromized, and borochromized samples after 100 h of oxidation at 850 °C and 950 °C.

**Figure 12:**
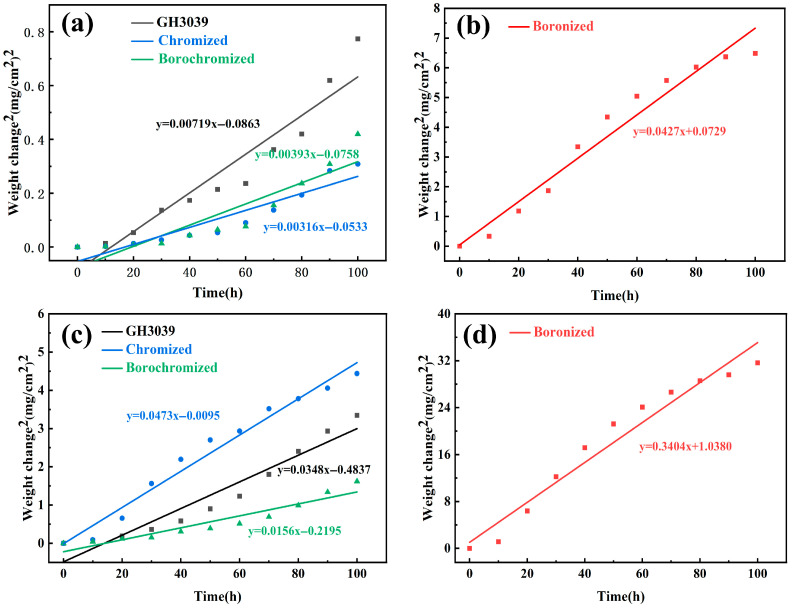
Parabolic oxidation kinetic fitting curves of GH3039, boronized, chromized and borochromized samples: (**a**,**b**) 850 °C; (**c**,**d**) 950 °C.

Figure 11 and Figure 12 show the mass-gain curves and the corresponding oxidation kinetic fitting results of the GH3039 alloy, boronized, chromized, and borochromized samples after oxidation at 850 °C and 950 °C for 100 h, respectively. At 850 °C, the boronized sample exhibited the highest oxidation mass gain, reaching 2.55 mg/cm^2^, whereas the mass gains of the GH3039 substrate, chromized, and borochromized samples were only 0.88, 0.56, and 0.65 mg/cm^2^, respectively. This indicates that both chromizing and borochromizing treatments can significantly improve the oxidation resistance of the substrate. To further quantitatively evaluate the high-temperature oxidation behavior of the samples, the oxidation mass-gain data were fitted using the parabolic oxidation kinetics theory proposed by Wagner [51], which can be expressed as:

(4) $${\left(\Delta m\right)}^{2}=\;K_{p}\times t+C$$

where Δm is the oxidation mass gain per unit area, t is the oxidation time, K_p_ is the parabolic oxidation rate constant, and C is the fitting constant. In general, a lower K_p_ value corresponds to better oxidation resistance. According to the fitting results, at 850 °C, the K_p_ values of the chromized and borochromized samples were significantly lower than those of the substrate and the boronized sample, indicating that the Cr-rich coatings effectively reduced the oxidation rate. Among all the samples, the chromized sample exhibited the lowest K_p_ value, followed by the borochromized sample and the GH3039 substrate, while the boronized sample showed the highest K_p_ value. This behavior is closely associated with the formation of a relatively thin and dense Cr_2_O_3_ protective film, which effectively suppresses the inward diffusion of oxygen and thereby enhances the oxidation resistance of the substrate. It should be noted that the R^2^ values for the GH3039 substrate, boronized, chromized, and borochromized samples at 850 °C were 0.9012, 0.9566, 0.8374, and 0.8660, respectively. The relatively lower correlation coefficients of the chromized and borochromized samples are mainly attributed to their slight oxidation and low total mass gain at this temperature. Under such conditions, only mild oxidation occurred on the coating surface, and the limited mass change amplified the influence of weighing errors and local non-uniform oxidation on the fitting results. In addition, these samples were still in the initial stage of oxide film formation and gradual densification, and their oxidation behavior had not yet fully conformed to the ideal parabolic law.

When the temperature increased to 950 °C, both the oxidation mass gain and the K_p_ values of all samples increased significantly, indicating that the oxidation reaction was markedly accelerated at elevated temperature. The R^2^ values of the oxidation kinetic fitting for the GH3039 substrate, boronized, chromized, and borochromized samples at 950 °C were 0.9281, 0.9566, 0.9722, and 0.9045, respectively, all of which are relatively high, suggesting good agreement between the experimental results and the parabolic kinetic relationship. Among all the samples, the boronized sample still exhibited the highest mass gain and the largest K_p_ value, with an oxidation mass gain of 5.63 mg/cm^2^, far exceeding those of the other samples. This is consistent with the cross-sectional observations, which show that the thickness of the boronized layer decreased markedly from 74.8 μm before oxidation to 27.1 μm after oxidation at 950 °C, accompanied by the formation of numerous cracks and pores within the coating. Moreover, the volatilization of B_2_O_3_ further weakened the compactness and long-term protective capability of the coating.

The chromized sample exhibited an oxidation mass gain of 2.11 mg/cm^2^ at 950 °C, which was higher than that of the GH3039 substrate (1.83 mg/cm^2^), and its K_p_ value also exceeded that of the substrate. This indicates that, during prolonged oxidation at high temperature, the protective effect of the single chromized layer deteriorated significantly due to the formation of a loose and porous structure in both the coating and the oxide scale, which disrupted the initially dense Cr_2_O_3_ film. In contrast, the borochromized sample exhibited the lowest oxidation mass gain, only 1.20 mg/cm^2^, together with the smallest K_p_ value (0.0156) among all the samples, demonstrating the best high-temperature oxidation resistance. This superior performance can be mainly attributed to the dense Cr-rich layer, which continuously supplies Cr for the formation of a protective Cr_2_O_3_ scale, effectively suppresses the inward diffusion of oxygen, reduces the tendency for pore formation, and preserves the structural integrity of the coating. In conclusion, the oxidation mass-gain curves are in good agreement with the kinetic fitting results, indicating that the borochromized coating combines a low oxidation rate with excellent long-term oxidation stability at high temperature, thereby exhibiting the best overall protective performance among all the tested samples.

**Figure 13:**
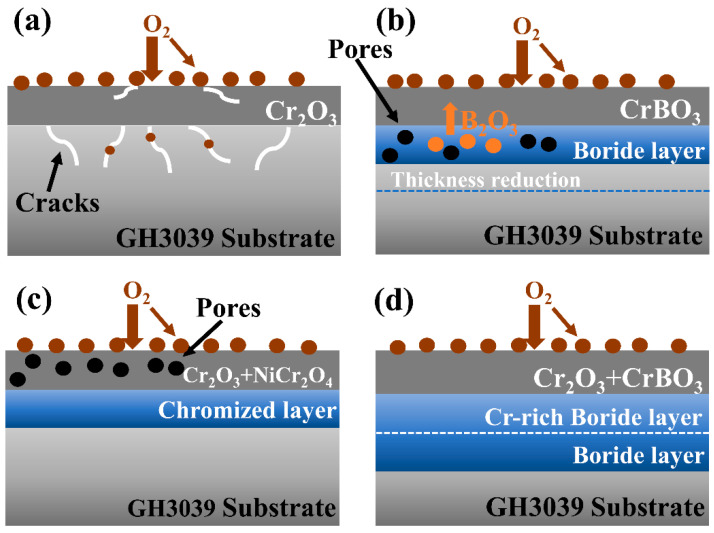
Schematic diagram of oxidation mechanism of GH3039 alloy and coating at 950 °C: (**a**) GH3039; (**b**) boronized coating; (**c**) chromized coating; (**d**) borochromized coating.

Figure 13 summarizes the oxidation mechanisms of GH3039 alloy, boronized, chromized and borochromized specimens at 950 °C. A Cr_2_O_3_ layer forms on the surface of GH3039 alloy. However, pores and cracks in the oxide layer and substrate act as rapid diffusion pathways for O, triggering internal oxidation of the alloy. The boronized coating can temporarily retard O diffusion to the substrate. Under prolonged high-temperature conditions, however, its thickness drastically reduces from 74.8 μm to 27.1 μm, accompanied by severe structural damage, making it incapable of long-term protection. Volume changes associated with oxidation products (NiO, Cr_2_O_3_) and volatile B_2_O_3_ induce pore formation, impairing the coating’s protective capacity. Meanwhile, Cr depletion in the coating results in the continuous formation of Cr_2_O_3_ film.

In the case of the chromized layer, the co-formation of Cr_2_O_3_ and NiCr_2_O_4_ spinel leads to a mismatch in thermal expansion coefficient between these phases. Additionally, the differences in diffusion coefficients between Ni and Cr further promote pore formation. The significant reduction in coating thickness and loss of dense Cr distribution weaken its protective performance. In contrast, the borochromized sample features a dense Cr-rich outer layer that continuously supplies Cr for Cr_2_O_3_ formation. This layer effectively inhibits outward Ni diffusion and reduces pore generation. After 100 h of oxidation, only the multiphase boride layer slightly thinning (from 72.8 to 63.9 μm), while the overall structure remains intact. This integrity enables the coating to provide long-term and stable oxidation protection for the substrate.

## 4. Conclusions

To simultaneously improve the friction and wear performance and high-temperature oxidation resistance of GH3039 alloy, borochromizing treatment was employed in this study, and the properties of boronized, chromized, and borochromized coatings were systematically compared. The main conclusions are as follows:

1. The boronized coating is composed of a Ni-rich top layer, a multiphase boride layer, and a diffusion layer. The chromized coating consists of a Cr-rich layer and a diffusion layer. The borochromized coating fabricated by boronizing followed by chromizing comprises a Cr-rich boride layer, a multiphase boride layer, and a diffusion layer, with a soft zone in hardness observed between the Cr-rich boride layer and the multi-phase boride layer.
2. Borochromizing treatment significantly enhances the surface hardness of GH3039 alloy, with a maximum value of 1918 HV_0.05_. A lubricating boric acid film forms on the coating during sliding, which effectively reduces the COF. The wear rate of the borochromized sample is decreased by 84.07% compared with the substrate, and the wear mechanism is transformed from adhesive wear-dominated for the substrate to abrasive wear-dominated for the borochromized coating.
3. Boronized, chromized, and borochromized coatings all improve the high-temperature oxidation resistance of GH3039 alloy, among which the borochromized coating exhibits the optimal performance. Its dense Cr-rich boride layer effectively restricts the inward diffusion of O and the outward diffusion of Ni, and provides sufficient Cr supply for the formation of a continuous Cr_2_O_3_ film. After high-temperature oxidation, the borochromized coating remains structurally intact, enabling long-term and stable high-temperature protection for the substrate.

## Figures and Tables

**Table 1:** Nominal chemical composition of GH3039 nickel-based alloy (in wt%).

Element	Content	Element	Content	Element	Content
Ni	Bal	Al	0.37–0.75	Mn	≤0.4
Cr	19–22	Ti	0.37–0.75	S	≤0.012
Mo	1.8–2.3	Fe	≤3.0	C	≤0.08
Nb	0.9–1.3	Si	≤0.8	P	≤0.02

**Table 2:** EDS composition analysis in Figure 1.

Element/at%	Ni	Cr	B	Fe
Spectrum 1	97.2	0.8	-	2.0
Spectrum 2	12.9	86.4	-	0.6
Spectrum 3	68.2	30.0	-	1.8

**Table 3:** EPMA composition analysis in Figure 2.

Element/at%	Ni	Cr	B	Fe
Spectrum 1	0.4	49.2	50.4	-
Spectrum 2	87.1	6.8	6.0	-
Spectrum 3	7.5	42.2	50.3	-
Spectrum 4	4.9	52.6	42.5	-

**Table 4:** EDS composition analysis in Figure 6.

Position	B (at%)	Ni (at%)	Cr (at%)	O (at%)	Si (at%)
Spectrum 1	-	68.1	22.1	8.6	1.2
Spectrum 2	-	43.9	14.2	33.9	8.0
Spectrum 3	56.5	41.9	0.2	1.3	-
Spectrum 4	59.2	40.0	0.3	0.5	0.1
Spectrum 5	-	34.8	13.8	42.4	8.9
Spectrum 6	-	41.8	15.8	35.6	6.7
Spectrum 7	-	70.5	23.9	4.9	0.8
Spectrum 8	50.2	15.3	32.6	1.6	0.3
Spectrum 9	58.7	38.7	0.3	1.8	0.4

## Data Availability

The original contributions presented in this study are included in the article. Further inquiries can be directed to the corresponding authors.
